# Effect of a sports-medicine–guided football program on physical fitness in adolescents: A controlled school-based trial

**DOI:** 10.1097/MD.0000000000048649

**Published:** 2026-05-22

**Authors:** Ke Shi, Yuelong Ye, Tianlun Zheng, Kaiyue Tang, Jing Bin, Liuxiang Wei, Lin Wang

**Affiliations:** a Guangzhou Vocational University of Science and Technology, Baiyun District, Guangzhou City, Guangdong Province, China; b College of Physical Education and Health, Guilin University, Guilin, Guangxi, China; c Department of Basic Courses, Chengxian College of Southeast University, Nanjing, Jiangsu, China; d Department of Physical Education and Art, Guilin No. 11 middle school Guilin, Guangxi, China; e Institutional Information and Address, Moscow State University of Sport and Tourism, Moscow, Russia.

**Keywords:** adolescent physical fitness, explainable artificial intelligence, injury risk prediction, machine learning ensemble, sports medicine intervention

## Abstract

Integrating artificial intelligence (AI) with sports medicine principles into school-based physical education may enhance physical fitness and reduce injury risk among adolescents. This study aimed to evaluate whether a 10-week sports-medicine-guided football curriculum augmented with explainable machine-learning feedback produces greater improvements in physical fitness and injury-prevention outcomes than a standard physical-education curriculum. A total of 195 healthy junior high school students (mean age 14.1 ± 0.7 years) participated in a controlled school-based intervention and were allocated to either an AI-supported sports-medicine curriculum (intervention) or a standard curriculum (control). Physical fitness outcomes, including lung capacity, estimated maximal oxygen uptake (VO_2_max), sprint performance, standing long jump, and middle-distance running performance (800 m girls/1000 m boys), were assessed before and after the 10-week program. Outcomes were analyzed using 2-way mixed analysis of variance to test Group × Time interactions. Injury risk prediction was supported by an explainable machine-learning ensemble model (CatBoost + Gradient Boosting) interpreted using SHAPley Additive explanations. Significant Group × Time interactions were observed for lung capacity (*P* < .01) and VO_2_max (*P* < .01), indicating greater improvements in the intervention group compared with controls. The intervention group also demonstrated superior improvements in sprint performance, middle-distance running, and lower-limb power (*P* < .05), while no significant between-group differences were observed for upper-body strength (*P* > .05). A school-based football curriculum integrating sports-medicine principles with explainable AI-driven feedback leads to significantly greater improvements in key physical-fitness outcomes than a standard physical-education curriculum. This approach directly supports individualized training adaptation and injury-risk awareness, offering a scalable model for enhancing adolescent health and safety in school physical education.

## 1. Introduction

Soccer is the world’s most popular team sport and has long been regarded as a vehicle for promoting physical health, motor development, and psychosocial skills in adolescents.^[1–3]^ In China, growing policy support and societal enthusiasm have contributed to the widespread integration of soccer into school curricula as part of national strategies to improve adolescent health and cultivate future athletic talent.^[4,5]^ The Healthy China 2030 initiative emphasizes comprehensive health education, shifting the focus from treatment to prevention and advocating for active, healthy lifestyles among children and adolescents.^[6–8]^ Within this context, school-based soccer programs are uniquely positioned to foster lifelong physical activity habits, enhance teamwork, and instill values of discipline and mutual respect.^[9,10]^

Despite these advances, challenges persist in designing and delivering scientifically sound, effective soccer curricula within schools. Research indicates that traditional approaches often lack standardization, rely on outdated training concepts, and insufficiently integrate modern principles of sports medicine and injury prevention.^[11,12]^ Furthermore, there is considerable variability in coach education, access to professional support, and the adequacy of infrastructure, leading to inconsistencies in the quality of school soccer instruction.^[13,14]^ Inadequate warm-up protocols, poor technical supervision, and a lack of individualized programming have all been identified as contributing to suboptimal outcomes and elevated risk of sports injuries among adolescent participants.^[15–18]^

Sports medicine provides a scientific foundation for physical education and youth sport by promoting safe, evidence-based training, optimizing performance, and minimizing injury risk.^[19–21]^ The integration of sports medicine principles, such as individualized exercise prescription, injury surveillance, health monitoring, and progressive overload, into school-based programs has been shown to enhance both short- and long-term health outcomes for students.^[22–24]^ For example, structured interventions that incorporate medical screening, regular assessment of physical fitness, and personalized feedback reduce the incidence of musculoskeletal injuries and support the holistic development of young athletes.^[25,26]^ However, large-scale implementation of such principles in school soccer curricula remains limited due to resource constraints and the shortage of trained medical or allied health professionals within educational settings.^[27,28]^

Recent advances in data science and artificial intelligence offer promising avenues to address these gaps. Machine learning algorithms are increasingly utilized in sports science to predict injury risk, monitor workload, and personalize training interventions.^[29–32]^ In elite football, machine learning (ML) models based on physiological, biomechanical, and historical injury data have achieved high accuracy in forecasting injuries, guiding return-to-play decisions, and optimizing load management.^[33,34]^ The application of explainable AI (XAI) techniques such as SHAP (SHapley Additive exPlanations) further enhances model transparency, supporting clinicians and coaches in interpreting predictions and making informed, individualized recommendations.^[35,36]^ Despite their promise, these data-driven technologies have been largely confined to professional sports settings, with limited translation to youth or school-based contexts.^[37,38]^

Meanwhile, there is growing recognition of the unique developmental challenges and vulnerabilities of adolescent athletes. Puberty is marked by rapid physical, cognitive, and psychosocial changes that affect response to training, injury risk, and overall well-being.^[39,40]^ Adolescents experience increases in height and body mass, shifts in muscle-to-fat ratio, and changes in neuromuscular control, all of which may impact both performance and injury susceptibility.^[41,42]^ Furthermore, balancing academic pressure, social dynamics, and sport participation can place additional strain on young students, necessitating comprehensive, multidisciplinary approaches to physical education.^[43,44]^

Emerging research from China and internationally highlights several persistent gaps and challenges in school soccer. These include insufficient emphasis on foundational movement skills, limited implementation of injury prevention strategies, and a lack of personalization in training load or feedback.^[45–48]^ Surveys of Chinese physical education programs reveal that injury rates remain unacceptably high and that many students lack basic knowledge regarding self-protection, warm-up, and safe exercise practices.^[49,50]^ Moreover, generic “one-size-fits-all” training approaches often fail to account for individual differences in growth, fitness, and prior injury history.^[51,52]^ Importantly, existing school-based football and injury-prevention programs have demonstrated measurable benefits for adolescent physical fitness. Prior studies report improvements in aerobic capacity, sprint performance, and lower-limb power following structured football-based or neuromuscular training interventions in school settings. These findings indicate that such programs can positively influence fitness development in adolescents. However, reported effects are often modest and heterogeneous, with outcomes varying widely across studies and student populations.

To address these issues, there is an urgent need for innovative, evidence-based curricula that combine the rigor of sports medicine with the scalability and precision of AI-driven technologies. Integrating machine learning into the design and implementation of school soccer programs enables dynamic monitoring of physical development, real-time injury risk assessment, and individualized feedback, capabilities that are otherwise resource-prohibitive in most educational settings.^[53,54]^ Such hybrid approaches hold promise for improving student safety, optimizing athletic development, and closing the gap between cutting-edge sports science and everyday school practice.^[55–60]^

Despite growing recognition of the importance of individualized training and injury prevention in youth sports, implementation remains challenging in real-world school settings. Many PE instructors, both in China and internationally, lack the advanced sports-medicine expertise, analytical tools, or technological support needed to design and monitor personalized exercise programs. Consequently, existing PE curricula often rely on uniform training plans that fail to account for individual variability in fitness level, biomechanics, and injury risk.

Advances in artificial intelligence and data-driven modeling now offer a practical solution to this gap, enabling real-time monitoring, adaptive training feedback, and interpretable injury-risk assessment. Integrating these technologies into physical education could bridge the gap between expert-level sports-medicine practice and everyday school implementation.

Therefore, this study hypothesizes that a school-based football program integrating sports-medicine principles with explainable AI feedback will produce significantly greater improvements in physical fitness and injury-prevention outcomes compared with a standard physical-education curriculum. This hypothesis is based on the rationale that individualized, data-informed guidance can better match each student’s physiological condition and workload tolerance, thereby optimizing performance while reducing injury risk.

## 2. Materials and methods

### 2.1. Study design and participants

This controlled, school-based educational intervention was conducted among 195 healthy, second-year junior-high-school students (mean age 14.1 ± 0.7 years; 96 boys and 99 girls) recruited through school-wide announcements and screened for eligibility.

Written informed consent was obtained from all participants and their guardians prior to enrollment. The study was approved by the Institutional Review Board and conducted in accordance with the Declaration of Helsinki.

**Figure 1.**
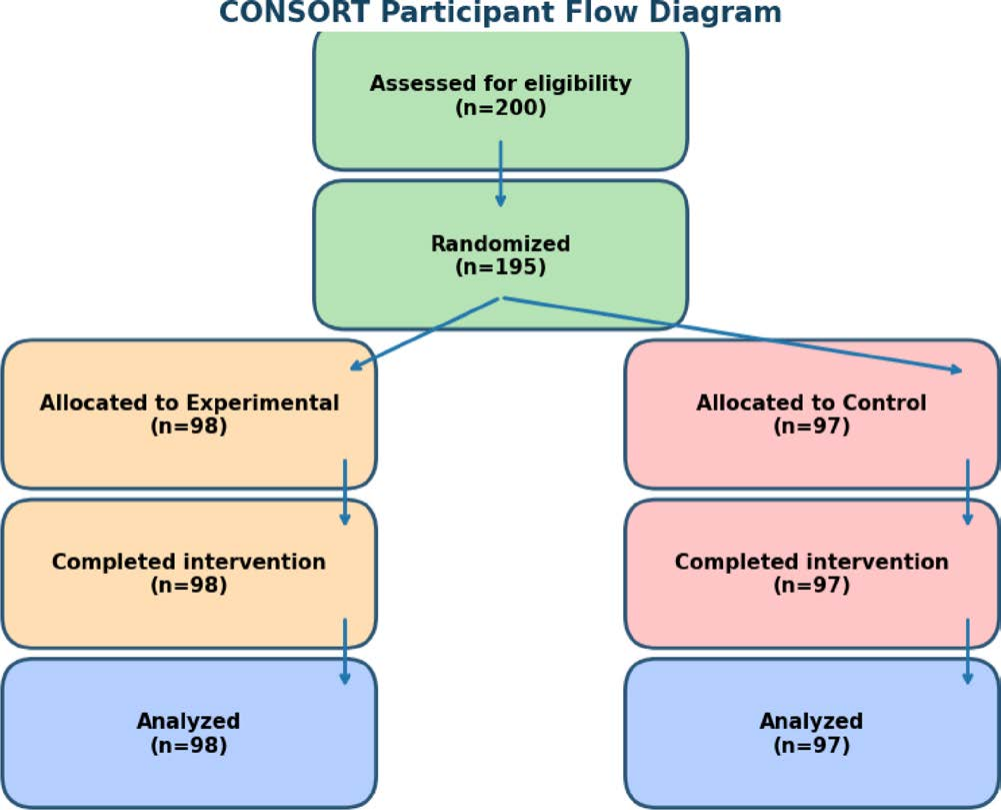
CONSORT participant flow diagram. Participant flow diagram illustrating the number of students screened, classified, allocated to intervention or control groups, completing the intervention, and included in final analyses. Reasons for exclusions and dropouts are indicated at each stage.

Allocation was performed at the class level to avoid contamination between groups. Two intact classes were assigned to either the intervention or control condition by school administration prior to study initiation. An intervention group (n = 98; 48 boys, 50 girls) and a control group (n = 97; 48 boys, 49 girls) ensuring a balanced gender ratio (Fig. 1). Baseline demographic and fitness characteristics were comparable between groups (Table 1).

**Table 1:** Baseline characteristics of study participants by group and gender.

Variable	Experimental group		Control group		*P*-value
	Male (n = 48)	Female (n = 50)	Male (n = 48)	Female (n = 49)	
Age (years)	14.2 ± 0.6	14.0 ± 0.7	14.1 ± 0.7	14.1 ± 0.6	.724
Height (cm)	156.4 ± 4.3	153.4 ± 2.4	157.5 ± 3.4	153.2 ± 2.1	.288
Weight (kg)	54.1 ± 8.7	47.3 ± 8.7	53.5 ± 9.6	47.6 ± 9.6	.342
BMI (kg/m^2^)	22.1 ± 2.6	20.1 ± 2.9	21.6 ± 2.9	20.3 ± 3.1	.418
Lung capacity (mL)	2485.4 ± 546.9	2471.2 ± 721.9	2131.7 ± 476.0	2539.5 ± 643.3	.091
VO_2_max (ml·kg^−1^·min^−1^)	46.92 ± 6.87	45.87 ± 3.75	48.32 ± 8.76	44.83 ± 4.43	.107
50 m sprint (s)	9.43 ± 0.43	9.21 ± 0.32	9.55 ± 1.22	9.19 ± 0.43	.255
Standing long jump (m)	1.76 ± 0.32	1.61 ± 0.23	1.75 ± 0.55	1.60 ± 0.19	.339
Pull-ups (count, M)	2.30 ± 1.40	—	2.12 ± 1.13	—	.462
Sit-ups (count, F)	—	33.4 ± 5.17	—	32.3 ± 11.2	.576
1000 m run (male, s)	334.9 ± 40.3	—	332.9 ± 38.9	—	.664
800 m run (female, s)	—	265.9 ± 36.0	—	275.4 ± 40.1	.312

Values are presented as mean ± standard deviation (SD). BMI = body mass index; VO_2_max = maximal oxygen uptake. *P*-values were obtained using independent-samples *t*-tests.

Values = mean ± SD. Exact 2-tailed *P*-values derived from independent-samples t tests comparing baseline experimental and control groups. No significant sex × group interaction was detected (all *P* > .05); thus, baseline data are reported by sex but analyzed collectively in subsequent mixed ANOVAs. Sex-related differences in baseline sprint performance should be interpreted in the context of pubertal timing, as girls typically mature earlier than boys during early adolescence.

### 2.2. Participants and eligibility

Students (second-year junior high) were eligible if they were physically healthy, cleared for participation in physical education, and free from conditions contraindicating outdoor exercise. Exclusion criteria included physician-diagnosed cardiopulmonary or neuromuscular disease, use of medications affecting cardiovascular, respiratory, or neuromuscular function, use of ergogenic supplements (e.g., caffeine pills, creatine, or pre-workout formulations), and recent musculoskeletal injury (<3 months).

Pubertal status was assessed using self-reported Tanner staging with guidance from parents or guardians. Students classified within Tanner stages II–IV were included, while those in stages I or V were excluded to minimize variability due to maturation extremes. Baseline habitual physical activity (minutes per week) and dietary or supplement use were also recorded.

### 2.3. Physical-fitness assessments and protocols

Testing was conducted in the morning, 48 to 72 hours after the last training session. Participants were instructed to avoid strenuous physical activity for 24 hours prior to testing, abstain from caffeine on the test day, and consume a light standardized breakfast at least 2 hours before assessment. Hydration was standardized according to recommended guidelines, with participants advised to consume approximately 5 to 7 mL/kg of water 3 to 4 hours before testing.

A standardized 10-minute dynamic warm-up consisting of light jogging, mobility exercises, and activation drills was performed prior to testing. The assessment sequence was fixed to minimize fatigue effects. Measurements were conducted in the following order: lung capacity (spirometry), estimated VO_2_max (20-m shuttle run), 50-m sprint, standing long jump, and middle-distance running performance (800 m for girls and 1000 m for boys).

A rest period of 3 to 5 minutes was provided between tests, with an extended recovery of 5 to 10 minutes before the middle-distance run.

### 2.4. Spirometry

Lung function was assessed using a portable spirometer (CONTEC SP10, China) in accordance with manufacturer guidelines and standardized school-based testing protocols. Each participant performed 3 maximal forced expiratory maneuvers from full inspiration while standing, with verbal encouragement provided by the assessor. A minimum rest period of 60 s was allowed between trials. The highest acceptable value of vital capacity (VC, mL) obtained across the trials was recorded for analysis. Trials were repeated if coughing, early termination, or poor effort was observed. Forced expiratory volume in 1 second (FEV_1_) was recorded for quality control but was not used as a primary outcome in the present analysis.

### 2.5. 20-m shuttle run test (VO_2_max estimation)

Aerobic capacity was evaluated using the 20-m shuttle run test following the National Students’ Physical Fitness and Health Standard protocol. Participants ran back and forth between 2 lines spaced 20 m apart, synchronized with audio signals that progressively increased in frequency. The test commenced at an initial speed of 8.5 km·h^−1^, with speed increments of 0.5 km·h^−1^ each minute. The test was terminated when the participant failed to reach the line in time on 2 consecutive occasions or voluntarily stopped due to fatigue. Total completed shuttles were recorded, and VO_2_max (mL·kg^−1^·min^−1^) was estimated using the standard National Students’ Physical Fitness and Health Standard prediction equation.

### 2.6. Assessment of habitual physical activity

Usual physical activity was recorded at baseline using a brief, structured self-report questionnaire completed by students with guidance from parents or guardians when needed. Participants reported the average weekly frequency and duration of organized sports participation, school-based physical-education classes, and leisure-time physical activity. Total habitual physical activity was calculated as minutes per week and used to characterize baseline activity levels and as a covariate in sensitivity analyses.

### 2.7. Intervention procedures

Intervention program. Delivered during regular PE periods (2×/week for 10 weeks), the program combined: standardized warm-up; football skill/game session; health literacy/injury-prevention mini-module; and individualized workload and risk feedback guided by the explainable ML module. Delivery was group-based (class size ∼ 45–50) with stations and teacher supervision.

### 2.8. Control group

The control group followed the school’s routine physical education curriculum, which included general calisthenics, a rotation of ball games (e.g., basketball, volleyball, and basic soccer activities), and light running exercises. This program did not incorporate structured sports-medicine principles, individualized load prescription, injury-prevention education, or machine-learning–based feedback. Training frequency and session duration were matched to those of the intervention group.

### 2.9. Machine learning–driven injury prediction

The injury-risk model was trained on adult football data but used calibrated probabilities (Platt scaling) and explainable outputs (SHAP) to support educational screening rather than clinical diagnosis. While feature attributions were consistent with known youth risk factors (exposure, prior injury), adolescent-specific external validation remains needed and is noted as a limitation.

**Figure 2.**
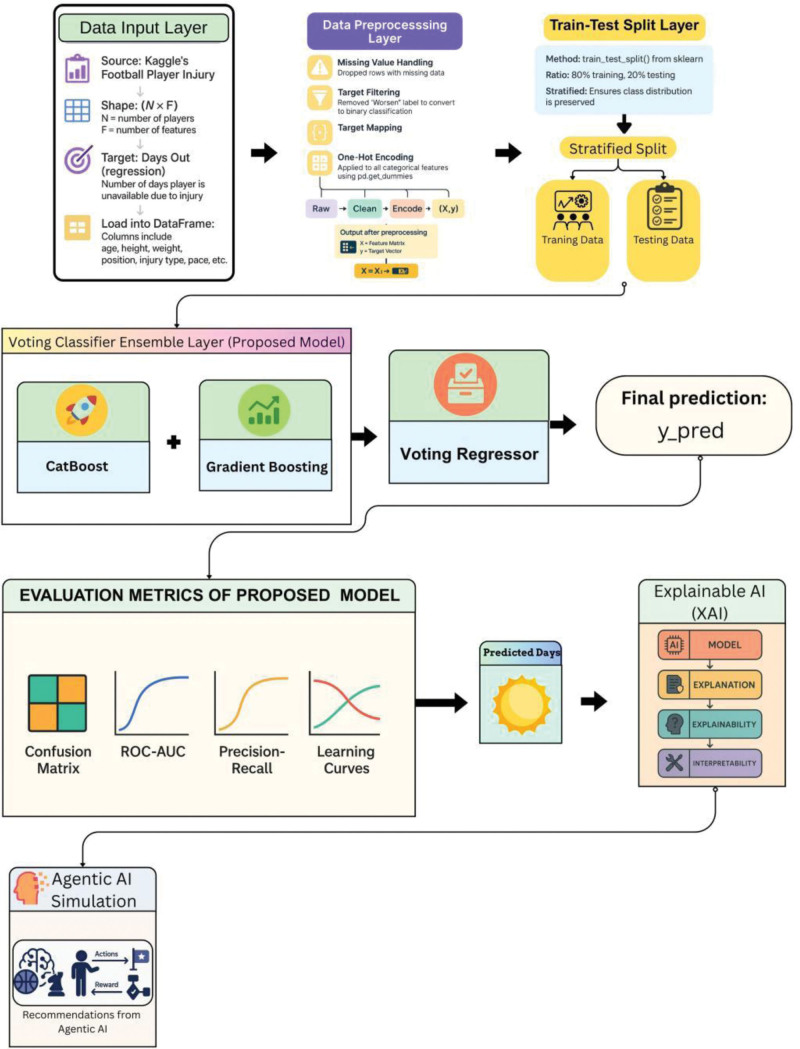
Architecture of the ensemble-based injury prediction system. Schematic overview of the ensemble-based injury prediction system workflow. The pipeline includes input of the football injury dataset, data preprocessing (handling missing values, encoding, normalization), stratified train-test split, model training using a Voting Regressor (CatBoost and Gradient Boosting), evaluation metrics computation, SHAP-based explainability, and an agentic AI feedback module providing individualized injury risk recommendations. Arrows indicate the sequence and data flow between modules.

Model training used a publicly available football injury dataset (Kaggle; n = 1301 player-season records) containing demographic, exposure, and injury history features. Preprocessing steps included removal of missing targets, one-hot encoding of categorical features, min-max normalization of continuous variables, and stratified 80:20 train-test split with 5-fold cross-validation (Fig. 2).

The final model was a Voting Regressor ensemble combining CatBoost (Yandex v1.2) and Gradient Boosting (scikit-learn v1.2), with equal weights:


$$\begin{array}{l} {\hat{y}}_{ensemble}=0.5\cdot f_{CatBoost}\left(X\right)+0.5\cdot f_{GBR}\left(X\right) \end{array}$$

Model performance was evaluated by mean absolute error (MAE), root mean squared error (RMSE), and R^2^:


$$\begin{array}{l} MAE=\frac{1}{n}\sum_{i=1}^{n}\left|y_{i}-{\hat{y}}_{i}\right| \end{array}$$

$$\begin{array}{l} RMSE=\sqrt{\frac{1}{n}\sum_{i=1}^{n}{\left(y_{i}-{\hat{y}}_{i}\right)}^{2}} \end{array}$$

$$\begin{array}{l} R^{2}=1-\frac{\sum_{i}{\left(y_{i}-{\hat{y}}_{i}\right)}^{2}}{\sum_{i}{\left(y_{i}-\bar{y}\right)}^{2}} \end{array}$$

Model interpretability was ensured using SHAP (SHapley Additive exPlanations), with SHAP values (ϕj) for feature j:


$$\begin{array}{l} \phi_{j}=\sum_{S\subseteq F\backslash \left\{\;j\right\}}\frac{\left|S\right|!\left(\left|F\right|-\left|S\right|-1\right)!}{\left|F\right|!}\left[f_{S\cup \left\{\;j\right\}}\left(x_{S\cup \left\{\;j\right\}}\right)-f_{S}\left(x_{S}\right)\right] \end{array}$$

An agentic AI feedback system generated individualized risk profiles and recommendations based on model and SHAP outputs.

### 2.10. Statistical analysis

Outcomes were analyzed using 2-way mixed-design ANOVA with Group (Intervention vs Control; between-subjects factor) and Time (Pre vs Post; within-subjects factor). The Group × Time interaction term was used to evaluate the effect of the intervention. When a significant interaction was observed, simple effects were examined using Bonferroni-adjusted pairwise comparisons.

Effect sizes were quantified using partial eta squared (η^2^), with values of 0.01, 0.06, and 0.14 interpreted as small, medium, and large effects, respectively. Assumptions of normality and homogeneity of variances were assessed prior to analysis. Sphericity was not applicable due to the presence of only 2 time points.

Sensitivity analyses were conducted using analysis of covariance on change scores, controlling for habitual physical activity (minutes per week), pubertal status (Tanner stage), and body mass index (BMI). These analyses yielded results consistent with the primary findings.

Within-group effect sizes were calculated using Cohen’s d with Hedges’ correction to account for small sample bias. These values are reported to describe the magnitude of within-group changes, whereas between-group differences are primarily interpreted based on the Group × Time interaction and corresponding partial η^2^ values.

Exact 2-tailed p-values are reported to 3 decimal places, with *P* < .001 reported where appropriate. Statistical analyses were performed using IBM SPSS Statistics (Version 26.0; IBM Corp., Armonk).

Machine learning analyses were conducted in Python (Version 3.10.12) using scikit-learn (Version 1.2.2) and CatBoost (Version 1.2). Data preprocessing and evaluation were performed using NumPy (Version 1.23.5) and pandas (Version 1.5.3). Model interpretability was implemented using the SHAP library (Version 0.41.0), and visualizations were generated using Matplotlib (Version 3.7.1).

## 3. Results

### 3.1. Baseline characteristics

Baseline demographic and physical fitness parameters, including age, height, weight, BMI, lung capacity, VO_2_max, 50m sprint, standing long jump, pull-ups (males), sit-ups (females), and running performance, were comparable between the intervention and control groups, with no significant differences (all *P* > .05).

### 3.2. Physical fitness outcomes

#### 3.2.1. Adherence and participant characteristics

All students completed ≥ 95% of scheduled sessions, with no injuries or dropouts during the 10-week program (Table 1). No significant differences were observed in sitting forward bend scores before and after the intervention in either group for both genders (*P* > .05).

#### 3.2.2. Lung capacity and VO_2_max

Two-way mixed ANOVA revealed a significant Group × Time interaction for lung capacity (*P* < .01), with a moderate-to-large effect size (partial η^2^ = 0.12). A significant Group × Time interaction was also observed for estimated VO_2_max (*P* < .01; partial η^2^ = 0.09), indicating that improvements in lung capacity were significantly greater in the intervention group compared with the control group.

Post hoc analyses showed significant pre–post increases in lung capacity and VO_2_max within the intervention group (all *P* < .01), whereas no significant changes were observed in the control group (Table 2).

**Table 2:** Lung capacity and maximal oxygen uptake before and after intervention.

Group	Gender	Lung capacity before (mL)	After (mL)	*P*-value	VO_2_max before (mL·kg^−1^·min^−1^)	After (mL·kg^−1^·min^−1^)	*P*-value
**Intervention group**	Male	2485.4 ± 546.9	2698.3 ± 498.5	**<.01**	46.92 ± 6.87	49.87 ± 4.05	**<.01**
	Female	2471.2 ± 721.9	2991.2 ± 632.3	**<.01**	45.87 ± 3.75	47.32 ± 2.48	**<.01**
**Control group**	Male	2131.7 ± 476.0	2093.6 ± 364.7	.412	48.32 ± 8.76	48.92 ± 3.96	.518
	Female	2539.5 ± 643.3	2559.5 ± 617.6	0.276	44.83 ± 4.43	45.25 ± 5.99	.331

Values are presented as mean ± SD. VO_2_max = maximal oxygen uptake. Within-group comparisons were conducted using paired t-tests. Significant *P*-values are highlighted in bold.

#### 3.2.3. Exercise performance (sprint, endurance, and lower-limb power)

Two-way mixed ANOVA revealed significant Group × Time interactions for sprint performance, middle-distance running performance, and standing long jump (all *P* < .05), indicating greater improvements in the intervention group compared with the control group (Table 3).

**Table 3:** Sprint, endurance, and lower-limb power results.

Group	Gender	50 m Sprint Before (s)	After (s)	*P*	Cohen *d*	Partial η^2^	Endurance Run (1000 m boys/ 800 m girls) Before (s)	After (s)	*P*	Cohen *d*	Partial η^2^	Standing Long Jump Before (m)	After (m)	*P*	Cohen *d*
**Experimental**	Male	9.13 ± 0.43	8.98 ± 0.88	.179	0.22	0.04	334.9 ± 40.3	319.2 ± 32.7	.005	0.43	0.09	1.76 ± 0.32	1.83 ± 0.18	0.087	.27
	Female	9.89 ± 0.32	9.01 ± 0.76	<.001	1.51	0.23	265.9 ± 36.0	258.4 ± 34.5	.139	0.21	0.05	1.61 ± 0.23	1.69 ± 0.27	0.030	.32
**Control**	Male	9.02 ± 1.22	9.01 ± 0.87	.894	0.01	—	332.9 ± 38.9	319.9 ± 36.7	.115	0.34	—	1.75 ± 0.55	1.76 ± 0.23	0.916	.02
	Female	9.93 ± 0.43	9.78 ± 0.46	.205	0.15	—	375.4 ± 40.1	284.8 ± 32.0	— †	—	—	1.60 ± 0.20	1.61 ± 0.12	0.671	.07

Values are presented as mean ± SD. Cohen *d* = effect size; η^2^ = partial eta squared.

Within the intervention group, females demonstrated a significant reduction in 50 m sprint time (from 9.89 ± 0.32 s to 9.01 ± 0.76 s; *P* < .001), and males showed significant improvements in 1000 m running performance (from 334.9 ± 40.3 s to 319.2 ± 32.7 s; *P* < .01). Females also improved in the 800 m run (from 265.9 ± 36.0 s to 258.4 ± 34.5 s; *P* < .05).

Standing long jump distance increased significantly in females (*P* < .05), while improvements in males were not statistically significant. No significant pre–post changes were observed in the control group (all *P* > .05) (Table 3).

#### 3.2.4. Upper body and core strength

Two-way mixed ANOVA revealed no significant Group × Time interaction for upper-body or core strength outcomes (pull-ups in boys; sit-ups in girls; *P* > .05). In addition, no significant main effect of Time was observed, indicating that these measures did not change significantly following the intervention in either the intervention or the control (Table 3).

### 3.3. Machine learning model performance

**Figure 3.**
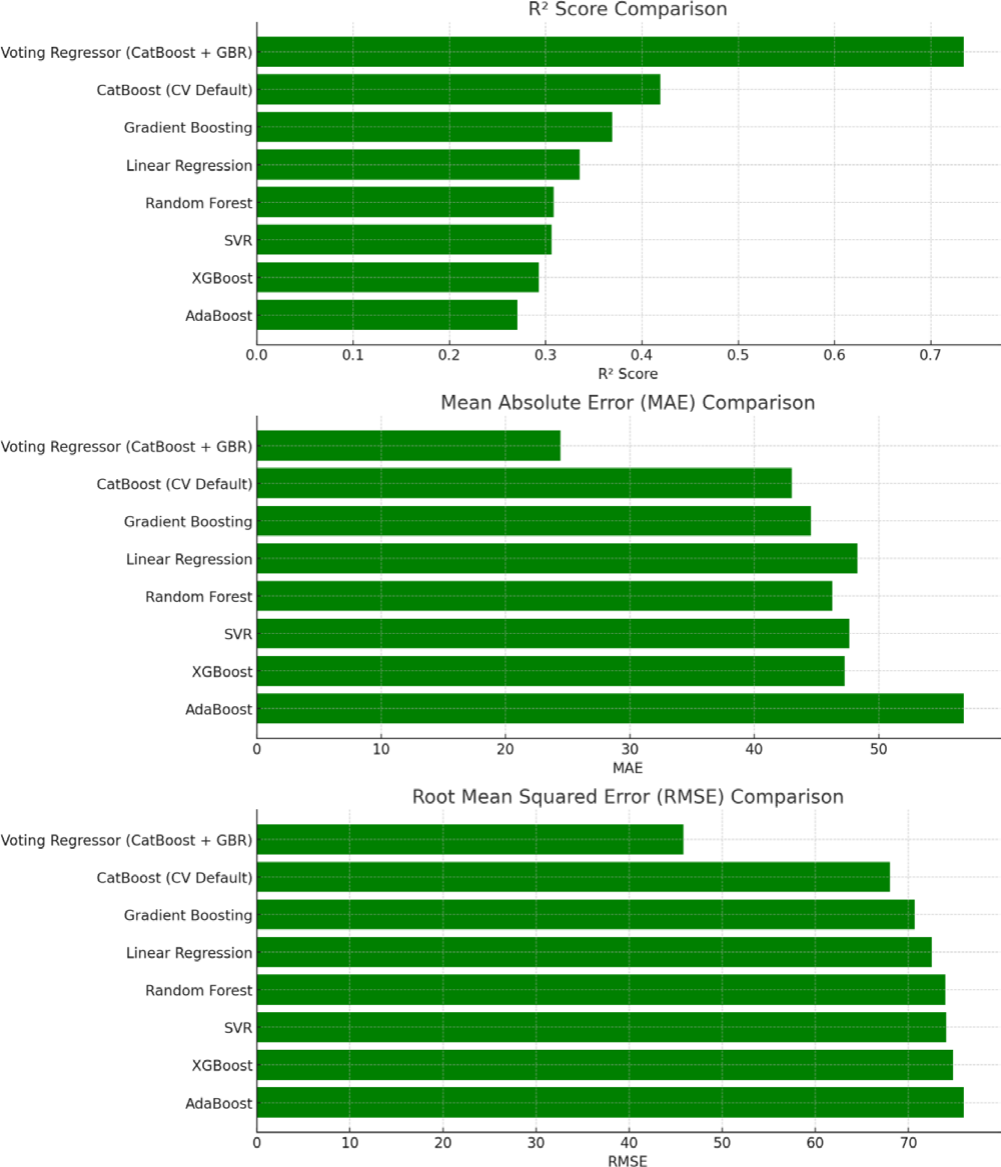
Bar plots comparing *R*^2^, MAE, and RMSE across regression models. Bar plots presenting the comparative performance of eleven regression models for predicting football player injury duration. Metrics shown include *R*^2^ score, mean absolute error (MAE), and root mean squared error (RMSE). The Voting Regressor (CatBoost + Gradient Boosting) demonstrated the highest *R*^2^ and lowest error rates, indicating superior predictive performance.

The ensemble-based Voting Regressor (CatBoost + Gradient Boosting) achieved the best performance for injury duration prediction, with a MAE of 24.41, RMSE of 45.84, and *R*^2^ score of 0.734 on the test set. Comparative results for all regression models are shown in Table 4 and illustrated in Figure 3.

**Table 4:** Comparative performance metrics of regression models for injury duration prediction.

Rank	Model	MAE	RMSE	*R*^2^ Score
1	Voting Regressor (CatBoost + GBR)	24.41	45.84	0.7343
2	CatBoost (Tuned, CV)	43.00	68.00	0.4525
3	CatBoost (Default CV)	43.00	68.00	0.4192
4	Gradient Boosting	44.55	70.65	0.3689
5	Voting Regressor (RF + SVR + XGB)	44.89	71.66	0.3507
6	Stacking Regressor	45.93	72.48	0.3357
7	Linear Regression	48.30	72.51	0.3353
8	Random Forest	46.27	73.96	0.3084
9	SVR	47.64	74.07	0.3063
10	XGBoost	47.26	74.78	0.2929
11	AdaBoost	56.85	75.95	0.2706

MAE = mean absolute error (average absolute difference between predicted and observed injury duration, expressed in days); RMSE = root mean squared error (square root of the mean squared prediction error, giving greater weight to large errors); *R*^2^ score = coefficient of determination, representing the proportion of variance in injury duration explained by the model. Lower MAE and RMSE values and higher R2 scores indicate better predictive performance.

**Figure 4.**
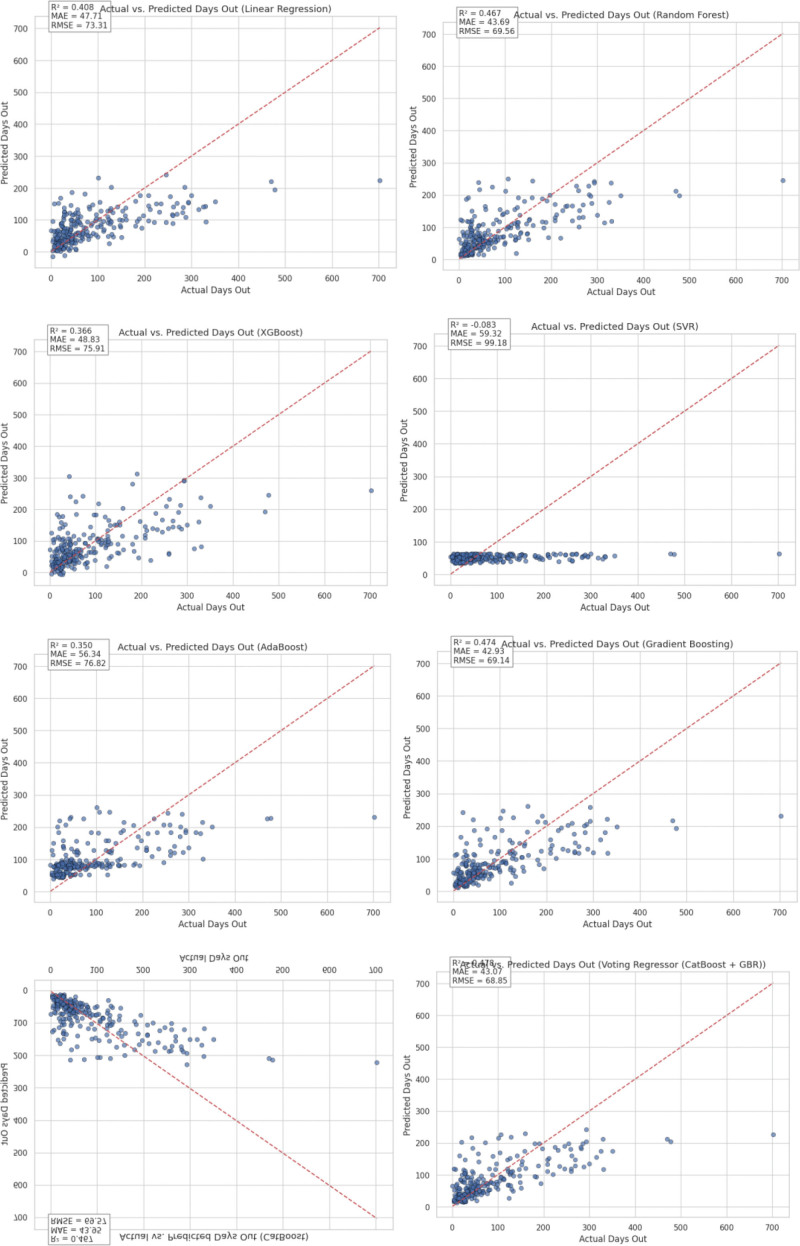
Scatter plots of actual versus predicted days out. Scatter plots comparing actual versus predicted injury recovery durations (days out) for selected regression models. The red dashed diagonal line indicates the ideal line of perfect prediction. Points clustered closer to the line reflect more accurate model performance.

**Figure 5.**
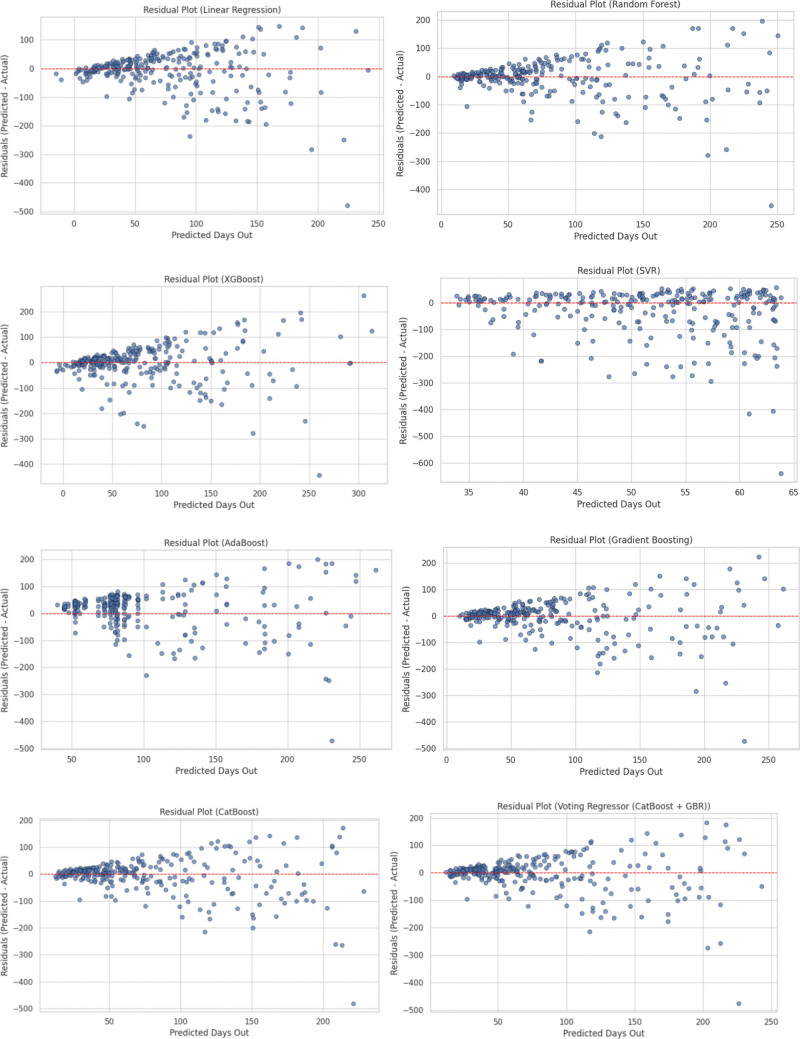
Residual distribution plots for all models. Residual distribution plots for all evaluated regression models, depicting the spread and symmetry of prediction errors (predicted minus actual days out). Narrower and more symmetric distributions around zero indicate improved model calibration and predictive consistency.

**Figure 6.**
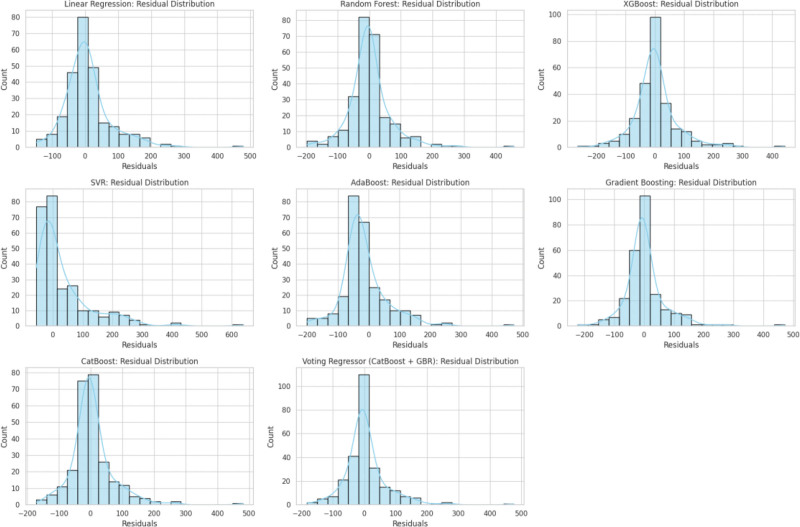
Residual distribution plots for all models, illustrating prediction error spread; the Voting Regressor shows the most centralized and symmetric residuals.

**Figure 7.**
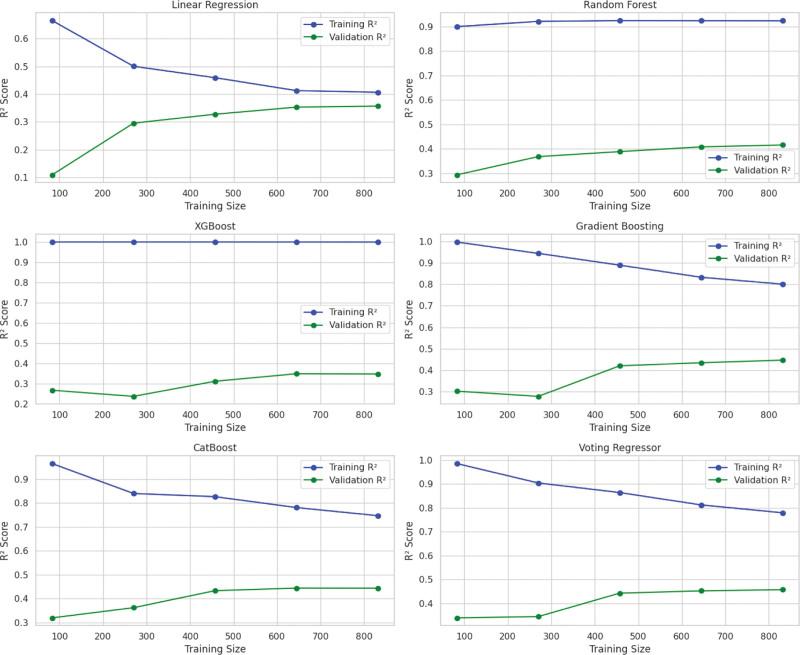
Learning curves depicting training and validation R^2^ scores across increasing dataset sizes for each model.

Model residuals were most centralized and symmetric for the Voting Regressor (Figs 4, 5, and 6), and learning curves for each model are depicted in Figure 7.

### 3.4. Model interpretability and agentic AI feedback

**Figure 8.**
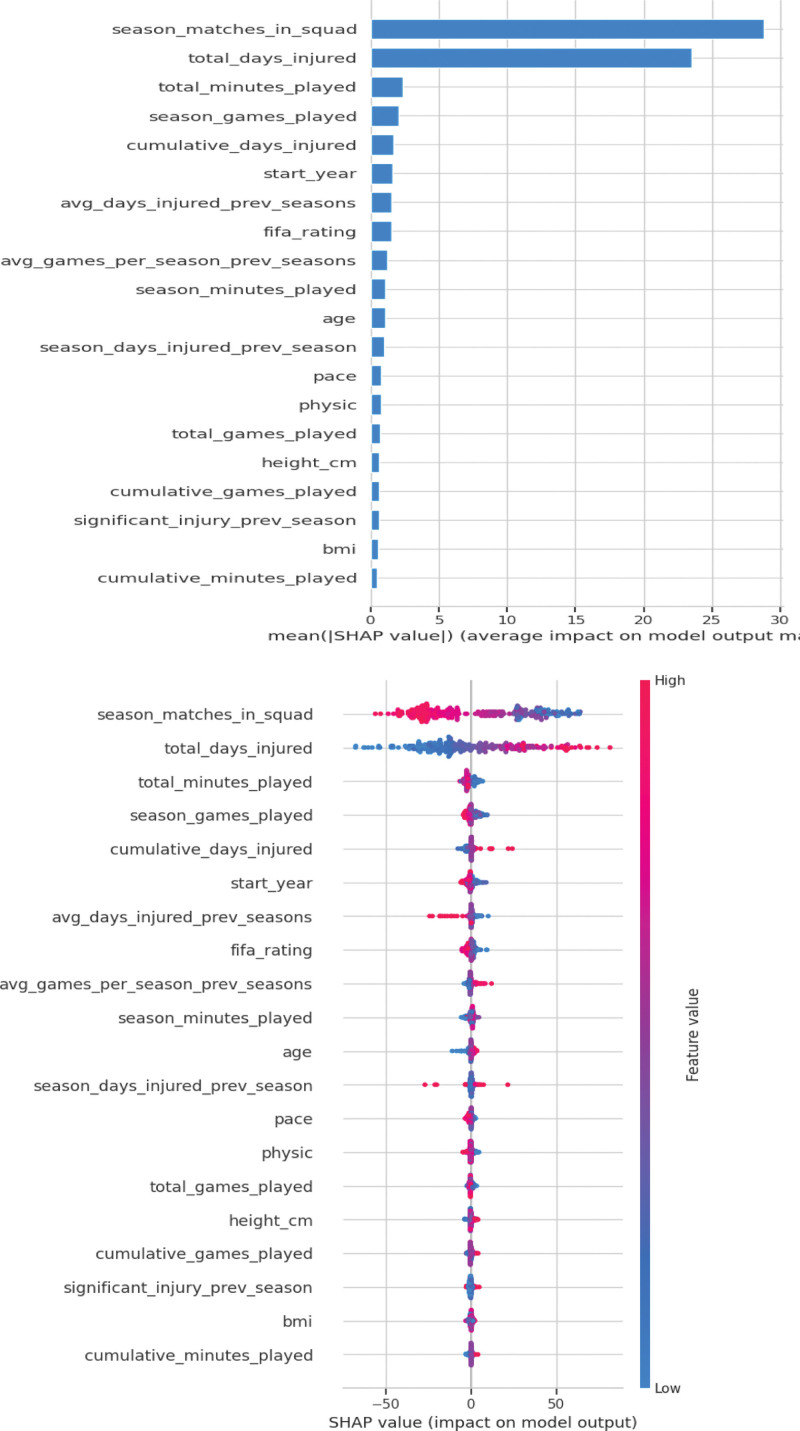
SHAP Beeswarm and Feature Importance Plots. SHAP (SHapley Additive exPlanations) beeswarm plot (left) and feature importance bar plot (right) for the final Voting Regressor model. The beeswarm plot visualizes individual feature effects on model output, while the bar plot summarizes mean absolute SHAP values, highlighting the most influential predictors of injury duration.

**Figure 9.**
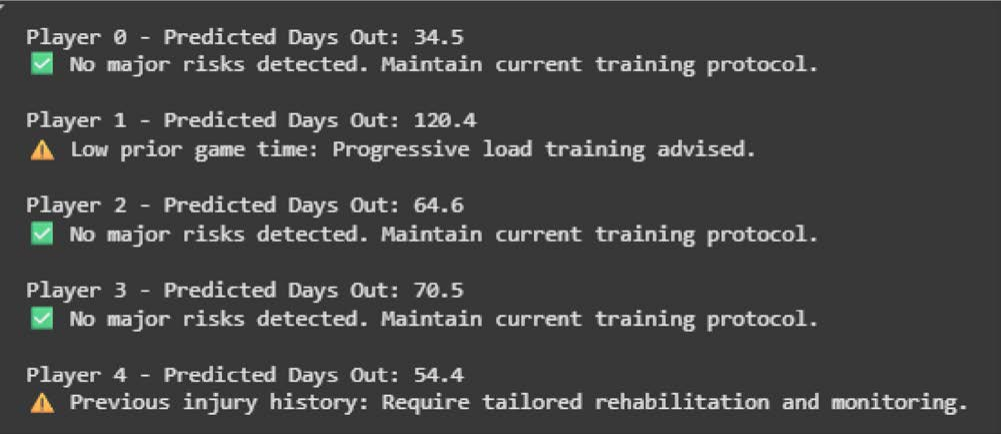
Sample outputs of agentic AI feedback. Examples of individualized feedback generated by the agentic AI module. Each output includes a predicted injury duration, key contributing risk factors, and personalized recommendations for training modification, rehabilitation, or monitoring.

The SHAP beeswarm plot (Fig. 8, left panel) illustrates both the magnitude and direction of each feature’s contribution to injury duration predictions. Higher values of cumulative injury days and total minutes played were associated with increased predicted injury duration, indicating that prior exposure and workload history were strong risk indicators. The feature importance plot (Fig. 8, right panel) summarizes the mean absolute SHAP values, confirming that exposure-related variables contributed most substantially to model output. These findings align with the study objective of developing a transparent injury-risk monitoring framework to support individualized training modification in school-based settings. The agentic AI module provided individualized feedback and training recommendations based on predicted injury duration (examples in Fig. 9).

## 4. Discussion

This study aimed to evaluate whether a school-based football program integrating sports-medicine principles with explainable AI feedback could enhance physical fitness and support injury-risk awareness compared with a control group curriculum.

The main findings showed that students participating in the integrated program demonstrated significant improvements in cardiopulmonary fitness and lower-limb performance compared with those following the standard curriculum. In particular, lung capacity and VO_2_max increased markedly, accompanied by gains in sprint and endurance performance. These results suggest that combining sports-medicine guidance with AI-driven feedback can optimize training adaptation, promote safer participation, and yield measurable fitness benefits in a school setting.

However, it is important to acknowledge that the superior gains observed in the intervention group may partly reflect a higher exercise intensity, total workload, or training volume, rather than the unique combination of AI and sports-medicine components alone. Although session frequency was matched between groups, the integrated program may have elicited greater effort or energy expenditure due to enhanced engagement and targeted load adjustments. Therefore, these improvements should be interpreted in light of the potential influence of training dose, which warrants further controlled evaluation.^[60–63]^

Our findings are consistent with prior research showing that structured, evidence-based PE interventions can improve aerobic capacity, motor skills, and musculoskeletal health in adolescents.^[61–63]^ These findings are consistent with previous studies demonstrating that structured football-based training improves aerobic capacity and neuromuscular performance in adolescents.^[29,53]^ The observed increases in VO_2_max and lung capacity are similar to those reported by Zou et al and Nikolaidis et al, who found that regular soccer training enhances respiratory and aerobic fitness in adolescents.^[29,53]^ The improvements in 50 m sprint and standing long jump further corroborate the value of multi-dimensional football curricula for speed and power development.^[51,52]^

A novel contribution of this study is the incorporation of ML-driven risk prediction and individualized recommendations into the PE environment. In professional football, recent research has leveraged ML algorithms and large-scale datasets to predict injury risk with increasing accuracy. For example, Sadurska and Piłka^[64]^ developed a football injury prediction model using an unbalanced dataset, emphasizing the importance of algorithm selection and feature engineering. Saberisani et al^[22]^ used GPS-derived workload and exposure data to predict injuries in Iranian professional footballers, demonstrating that ML approaches can identify key risk indicators and guide intervention. Freitas et al^[41]^ and Rossi et al^[45]^ both confirmed that ML models can effectively predict noncontact injuries and future injury episodes using physical, training, and exposure data in professional and semiprofessional contexts.

Most previous studies, however, focus on elite adult athletes and seldom address the unique developmental and educational needs of adolescent students.^[56]^ Translating these models into school-based practice presents distinct challenges, including the need for explainable, user-friendly outputs and attention to individual growth and adaptation. The current study addresses this gap by integrating a transparent Voting Regressor ensemble (CatBoost and Gradient Boosting) and SHAP-based interpretability, generating individualized feedback for both students and teachers.

Bartels et al^[35]^ further highlight the complexities of practical ML deployment in football, particularly the necessity for interpretable algorithms and clear communication of results to non-experts. Our use of SHAP values to pinpoint major risk contributors, such as cumulative injury days and prior exposure supports the practical applicability and pedagogical value of explainable AI in real-world school sport.

In the present study, SHAP analysis revealed that cumulative injury days, total minutes played, and season matches in squad were the most influential predictors of injury duration within the ensemble model. The predominance of exposure-related variables suggests that workload accumulation and prior injury history remain central determinants of predicted injury duration. Although the dataset was derived from professional football players, the prominence of exposure metrics is consistent with established injury epidemiology frameworks emphasizing cumulative load and recovery balance. In the school-based context, these findings highlight the importance of monitoring participation volume and prior injury history when designing football curricula for adolescents. Importantly, the machine-learning model was not used as a clinical diagnostic tool but rather as an educational risk-awareness system, supporting individualized workload modification and reinforcing safe training practices.

The superiority of the ML-augmented, sports medicine–guided curriculum over control is likely due to the combined effects of evidence-based training progression, continuous monitoring, and individualized feedback. Consistent with established sports medicine principles, gradual load progression, structured warm-up/cool-down, and the inclusion of health literacy content contributed to injury prevention and improved adaptation.^[25–27]^ The ML component empowered teachers to recognize at-risk students, modify training in real time, and foster a culture of self-monitoring and proactive health management among students, an advance not previously described in school settings.

The greater improvements observed in intervention group may reflect differences in program structure, participant engagement, or exercise intensity promoted by the individualized feedback framework. Although the intervention may have elicited differences in exercise intensity or engagement, objective indices of internal or external training load (e.g., heart rate, Borg RPE, TRIMP, or energy expenditure) were not collected. As such, any inference regarding training intensity should be interpreted cautiously.

These findings have important implications for both research and practice. Scientifically, this study provides real-world evidence that integrating explainable ML into PE is feasible and effective for injury prevention and performance enhancement. The approach is scalable, potentially reducing reliance on medical personnel and enabling broader implementation in resource-limited schools. Practically, this strategy aligns with public health objectives such as China’s Healthy 2030 initiative, by fostering physical literacy, reducing sports injuries, and supporting long-term athletic development at the population level.^[8,38]^

Several physiological, behavioral, and pedagogical mechanisms may explain the observed benefits. First, regular soccer training promotes cardiovascular and respiratory adaptation, neuromuscular coordination, and musculoskeletal strength in growing adolescents.^[40,42]^ Second, the curriculum’s focus on health education and injury awareness likely enhanced self-protection behaviors, as found in other school-based health interventions.^[50]^ Third, individualized, ML-driven feedback may increase student engagement, adherence, and self-efficacy by providing tangible, personal insights and facilitating goal setting.^[37,58]^ The use of SHAP-based explainability allowed instructors and students alike to understand the “why” behind each risk prediction, increasing trust and utility.

These results indicate moderate-to-large effect sizes in key aerobic and power indicators, particularly among girls, supporting the efficacy of the integrated program.

Notably, upper-body strength measures did not demonstrate significant changes following the 10-week intervention. This likely reflects the football-specific emphasis of the curriculum, which primarily targets aerobic capacity, running performance, and lower-limb explosive power rather than upper-body muscular development. The training stimulus may have been insufficient in volume or specificity to induce measurable adaptations in pull-up or sit-up performance. In adolescents, neuromuscular adaptations in upper-body musculature may require longer intervention duration or targeted resistance training to produce detectable changes.

### 4.1. Limitations and future perspectives

Several limitations should be considered when interpreting the present findings. First, although participants were screened for health status and training background, habitual physical activity outside the school program and dietary intake were not objectively monitored, which may have influenced the magnitude of adaptation. Second, maturational status was self-reported rather than clinically verified; therefore, minor differences in pubertal stage may have affected physiological responses. Third, although both groups trained with the same weekly frequency, exercise intensity and actual training volume were not continuously quantified, limiting precise comparison of workload exposure between programs. Environmental factors such as sleep and hydration were standardized only through general guidance rather than objective monitoring. Blinding was also limited. Although outcome assessors were independent of curriculum delivery and data were coded, full participant blinding was not feasible due to the nature of the school-based intervention, which may have introduced performance-related bias. In addition, the absence of a true non-exercise control group limits the ability to isolate the independent effects of exercise exposure from curriculum design. Future studies incorporating an inactive comparison group may better distinguish the relative contributions of training volume and program structure. Finally, although the AI-based injury-risk model was calibrated for adolescent application, it was originally derived from adult football datasets and therefore requires further external validation in school-aged populations to confirm generalizability. Future research should incorporate objective monitoring of heart rate and training load, validated indices of biological maturation, and standardized dietary assessment. Longitudinal follow-up is also warranted to determine whether improvements in fitness and injury-risk awareness are sustained beyond the structured intervention period.

### 4.2. Clinical and practical applications

The study demonstrates that the integration of explainable machine learning and sports medicine principles in school PE programs is not only feasible but beneficial for student health and safety. This approach provides a replicable model for data-driven, individualized injury prevention and performance optimization in youth sports, supporting current trends in personalized education and digital health.

### 4.3. Conclusion

This study demonstrates that integrating sports-medicine principles with explainable AI feedback into a school-based football curriculum significantly improved aerobic capacity (VO_2_max), lung capacity, middle-distance running performance, and lower-limb explosive power among adolescents. However, upper-body strength measures did not show significant changes following the 10-week intervention. The inclusion of individualized, data-driven feedback supported injury-risk awareness and workload monitoring rather than direct injury prevention. Overall, this approach offers a structured and scalable framework for enhancing fitness development and promoting safe participation in school physical education settings.

## Acknowledgments

The authors gratefully acknowledge the support of the participating schools, students, and staff who made this study possible. No professional writing assistance was used in the preparation of this manuscript.

## Author contributions

**Conceptualization:** Yuelong Ye, Kaiyue Tang.

**Formal analysis:** Yuelong Ye.

**Investigation:** Tianlun Zheng, Kaiyue Tang, Jing Bin, Liuxiang Wei.

**Methodology:** Tianlun Zheng, Kaiyue Tang, Jing Bin, Liuxiang Wei.

**Writing – original draft:** Ke Shi, Lin Wang.

**Writing – review & editing:** Ke Shi, Kaiyue Tang, Lin Wang.
